# Association between LRP5 polymorphism and bone mineral density: a Bayesian meta-analysis

**DOI:** 10.1186/1471-2350-9-55

**Published:** 2008-06-27

**Authors:** Bich NH Tran, Nguyen D Nguyen, John A Eisman, Tuan V Nguyen

**Affiliations:** 1 Bone and Mineral Research Program, Garvan Institute of Medical Research, St Vincent's Hospital, Sydney, Australia; 2 Faculty of Medicine, The University of New South Wales, Sydney, Australia

## Abstract

**Background:**

The low-density lipoprotein receptor-related protein 5 gene (LRP5) was identified to be linked to the variation in BMD in high bone mass pedigrees. Subsequent population-based studies of the association between the LRP5 gene and BMD have yielded conflicting results. The present study was aimed at examining the association between LRP5 gene and BMD by using meta-analysis.

**Methods:**

A systematic electronic search of literature was conducted to identify all published studies in English on the association between LRP5 gene and osteoporosis-related phenotypes, including bone mineral density and fracture. BMD data were summarized from individual studies by LRP5 genotype, and a synthesis of data was performed with random-effects meta-analyses. After excluding studies on animal and review papers, there were 19 studies for the synthesis. Among these studies, 10 studies used the rs3736228 (A1330V) polymorphism and reported BMD values.

**Results:**

The 10 eligible studies comprised 16,705 individuals, with the majority being women (n = 8444), aged between 18 – 81 years. The overall distribution of genotype frequencies was: AA, 68%, AV and VV, 32%. However, the genotype frequency varied significantly within as well as between ethnic populations. On random-effects meta-analysis, lumbar spine BMD among individuals with the AA genotype was on average 0.018 (95% confidence interval [CI]: 0.012 to 0.023) g/cm^2 ^higher than those with either AV or VV genotype. Similarly, femoral neck BMD among carriers of the AA genotype was 0.011 (95%CI: 0.004 to 0.017) g/cm^2 ^higher than those without the genotype. While there was no significant heterogeneity in the association between the A1330V polymorphism and lumbar spine BMD (p = 0.55), the association was heterogeneous for femoral neck BMD (p = 0.05). The probability that the difference is greater than one standard deviation was 0.34 for femoral neck BMD and 0.54 for lumbar spine BMD.

**Conclusion:**

These results suggest that there is a modest effect of the A1330V polymorphism on BMD in the general population, and that the modest association may limit its clinical use.

## Background

Bone mineral density (BMD) is a primary predictor of osteoporotic fracture [1], and is used as a surrogate definition of osteoporosis [2]. Several epidemiological studies have consistently demonstrated that each standard deviation (SD) lowering in BMD is associated with an approximately 2-fold increase in fracture risk [3]. This strength of association is equivalent to or even stronger than the association between serum cholesterol and cardiovascular events [4], or between blood pressure measurements and risk of stroke mortality [5]. BMD changes with age, with peak levels been reached between the age of 20 and 30 and then decreasing during the later decades of life. Any BMD level -2.5 standard deviations or more below the young normal average level is classified as osteoporosis [6].

Extensive evidence from twin studies and family-based studies have suggested that between 60% and 82% variance of BMD is attributable to genetic factors [7,8]. During the past two decades, it has become clear that many genes contribute to the variation in BMD in the general population; however, the localization of specific genes has not been always successful, due to on-going conflicting and contradictory findings [9].

A linkage analysis of a pedigree from a proband with the osteoporosis-pseudoglioma syndrome (OPS), a disorder characterized by severely low bone mass and eye abnormality, identified a locus on chromosome 11 linked to variation in BMD in the pedigree [10]. The linkage between the region and BMD was reported independently from a genome-wide linkage analysis of an extended family with 22 members among whom 12 had very high bone mass [11]. In follow-up studies using the positional candidate approach both research groups found that the gene encoding the low-density lipoprotein receptor-related protein 5 (LRP5) was linked to both OPS and high bone mass [12-14]. Six novel mutations in LRP5 among 13 confirmed polymorphisms have also been associated with different conditions with increased BMD [15].

Since the identification of the LRP5 gene, several population-based association studies have examined the association between LRP5 polymorphisms and normal variation in BMD [14,16-24]. However, these studies have yielded contradictory results, with some studies showing a significant association, while others did not. Thus, the role of LRP5 gene in the regulation of BMD in the general population has not been clear. In the presence of inconsistent findings, a systematic review by compiling all available data and synthesizing them into a coherent summary may provide a more reliable conclusion about the association between LRP5 polymorphisms and BMD. The present study was aimed at using the Bayesian approach to summarize the overall effect of LRP polymorphisms on BMD variation in different populations.

## Methods

### Literature search

A systematic search the literature was carried out by using electronic databases including Pubmed, Ovid (from 2001 to March 2008), and the Cochrane Controlled Trials Registered from 1960 to March 2004. The language was limited to English. The keywords used for this search were "LDL receptor-related protein 5 gene OR LRP5 gene" concatenated with "osteoporosis OR bone mineral density OR bone density OR BMD", and "fracture*". Two reviewers (BT and NN) identified eligible articles for which the abstracts were recorded. Then, if the abstract was consistent with the inclusion criteria, the full article text was obtained. The inclusion criteria were (a) original papers; (b) population-based association study with BMD being the outcome; and (c) adult men or women (aged 18+ years). The exclusion criteria were: (a) animal studies; (c) family studies; (c) review papers; and (d) studies on children or adolescents.

The full texts of all potentially relevant papers were obtained and three reviewers (BT, NN and TN) independently checked for data consistency. If more than one paper with the same data was identified, only the one that contained the original data were included. For studies in which BMD measurements were not presented as mean and SD for each genotype, we contacted the authors to request the data using a formatted collection form. For each study, relevant data including details of study design, study duration, gender, BMD measurements, LRP5 polymorphisms, inclusion and exclusion criteria, and incidence of fractures were extracted. As there have been very few studies on an association between LRP5 polymorphism and fracture [16], the primary outcome in this meta-analysis was BMD (Table 1).

**Table 1:** Characteristics of individual studies

Study	Study design	Ethnicity	Age (mean or range)	BMD measurement	Sex	Frequency of A1330V genotypes			Frequency of AA
							
						AA	AV	VV	
Koh, 2004 [23]	CS	Asian	25.6	LS + FN	Men	161	51	7	0.74
Mizuguchi, 2004 [37]	CC	Asian	54.2	LS	Women	129	114	11	0.51
Koller, 2005 [24]	CS	Caucasian	20 – 50	LS + FN	Women	833	416	52	0.64
Zhang, 2005 [38]	C	Asian	60.1	LS + FN	Women	440	192	15	0.68
van Meurs, 2006 [39]	C	Caucasian	≥ 55	LS + FN	Men^a^	895	643	54	0.56
		Caucasian			Women	2766	939	76	0.73
Ezura, 2007 [18]	CS	Asian	64.6	LS	Women	178	174	35	0.46
Saarinen, 2007 [40]	CS	Caucasian	18 – 21	LS + FN	Men	215	20		0.91
Giroux, 2007 [20]	CS	Caucasian	53.3	LS + FN	Women	1452	622		0.70
Grundberg, 2007 [21]	CS	Caucasian	69 – 81	LS + FN	Men	2114	620	33	0.76
	CS	Asian	> 65	LS + FN	Men	1067	487	70	0.66
	CS	Caucasian	18 – 20	LS + FN	Men	806	216	23	0.77
Brixen, 2007 [36]	CS	Caucasian	20 – 30	LS	Men	589	170	20	0.76

CS = cross-sectional study, CC = case-control study, C = cohort study; LS = lumbar spine, FN = femoral neck.

^a^The distribution of genotypes was not consistent with the Hardy-Weinberg's equilibrium law (p < 0.0001).

### Data synthesis and analysis

In each study, the outcome data (BMD) were extracted and summarized by genotype. The effect size for each study was the difference in BMD between genotypes (denoted *d*_*i*_). The aim was to estimate an overall effect or weighted mean difference (WMD) in BMD between genotypes (denoted by *d*). This was done by both traditional (fixed-effects and random-effects models) meta-analysis [25], which have been described elsewhere [26] and fully Bayesian method [27,28]. Briefly, each *d*_*i *_is assumed to be normally distributed with a "true" but unknown effect size *θ*_*i *_and a within-study variance $\sigma_{i}^{2}$. Furthermore, the collection of *d*_*i *_across studies is assumed to follow a normal distribution with unknown mean *θ *and variance *τ*^2^. Thus, *θ *is the overall WMD in BMD between genotypes across studies and *τ*^2 ^is the between-study variance. The classical fixed-effects method of meta-analysis assumes that *τ*^2 ^= 0, whereas the classical random-effects method recognizes the possibility of heterogeneity of study-to-study variation (i.e., that *τ*^2 ^could be difference from 0). All parameters of the classical fixed and random-effects model were estimated by the inverse variance weighting method as implemented by the "meta" package within the R language [29].

In contrast to the traditional random-effects model where the parameters *θ*, *σ*^2 ^and *τ*^2 ^are assumed to be fixed, in Bayesian random-effects model, $\sigma_{i}^{2}$ and *τ*^2 ^are assumed to be random variables. Furthermore, the Bayesian approach allows incorporate the existing data into the present analysis; therefore, the effect sizes of association between LRP5 variants and bone mineral density from a recent large-scale study were included in the analysis [30].

Fully Bayesian analysis refers to the use of external prior information, which must be specified for *θ *and *τ*^2 ^in the estimation of the overall effect size. In this analysis, the prior distribution for *τ*^2 ^was assumed to be uniformly distributed with parameters (0, 10) to recognize the uncertainty of effect sizes. The prior distribution for *θ *was given as a normal distribution of mean 0 and variance of 10000 to reflect the fact that the knowledge of effect sizes was vague. This is also considered a "referent prior", in the sense that it reflects the equal effect of genotypes in BMD variations. The estimation of model parameters was performed by the MCMC technique with the WinBUGS program [31].

The heterogeneity of effects across studies was assessed by computing the Cochran's *Q *statistic [32] and the coefficient of inconsistency (*I*^2^), as described by Higgins et al [33]. Funnel plots were performed to identify any possible evidence of publication bias [34,35]. Finally, recursive cumulative meta-analysis was also performed to examine whether the magnitude of effect changes markedly with sample size. In this analysis, each smaller size study was considered as an informative step, in which evidence was updated by larger sample size studies published in the interim.

## Results

### Characteristics of studies

**Figure 1:**
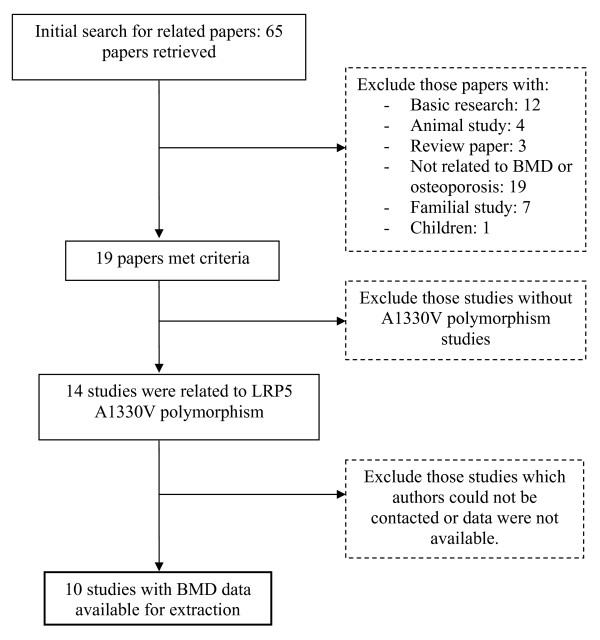
Summary of search strategy and result.

The electronic search yielded 65 papers on the association between LRP5 and osteoporosis-related phenotypes; however, only 19 met the inclusion criteria (Figure 1). Among the 22 SNPs used in various studies, the following 10 SNPs were more common (in order of the frequency of studies): rs3736228, rs4988321, rs41494349, rs2277268, rs2306862, rs556442, rs17149104, rs11574422, rs545382, rs4988319. Fourteen studies examined the association between the SNP rs3736228 (A1330V polymorphism, alanine-to-valine substitution at position 1330 in exon 18) within the LRP5 gene and BMD or fracture in human. Among the 14 studies, 8 papers reported actual BMD data by genotype. Corresponding authors of the 6 remaining papers were contacted with a data collection form, however, only 2 responded. The three studies that were not included in this analysis found no significant association between the SNP rs3736228 (referred as SNP A1330V in the article) and BMD. Eventually, data from 10 studies [18,20,21,23,24,36-40] were included in the traditional analysis of association with BMD. In a recent large-scale analysis of the association between LRP5 polymorphisms and BMD or fracture in Caucasian individuals, the results were only shown effect sizes of the association [30]; therefore, this study was only able to be incorporated in the Bayesian approach.

Five studies were conducted on Asian populations (i.e., Chinese, Japanese, and Korean) [18,21,23,24,37,38], with a total sample size of 3131 individuals and 6 studies were on Caucasian populations with 13,574 individuals [20,21,24,36,39,40]. Approximately 80% of participants were women. The average age of all participants was 60 years (range: 18 to 81 years).

### Distribution of LRP5 genotypes

There was high variability in the genotypic distribution within and between populations. For example, within the Asian populations, the relative frequency of the A1330V AA genotype ranged between 46% in Japanese women [18] to 68% in Chinese women [38] and 64% in Caucasian women [24]. However, in a study among Korean men, the frequency of AA genotype was 74% [23]. In Caucasian populations, the A1330V AA genotype was found in 56% of Dutch men [39], which was significantly lower than that in Finnish men 91% [40] (Table 2). The frequency of AA genotype in Swedish men was around 76% [21].

**Table 2:** Summary of BMD data by A1330V genotype

First author, year	Gender	Femoral neck BMD		Lumbar spine BMD	
		
		AA	AV/VV	AA	AV/VV
Koh, 2004 [23]	Men	1.07 (0.15)	1.03 (0.15)	1.20 (0.18)	1.21 (0.13)
Mizuguchi, 2004 [37]	Women	-	-	0.81 (0.16)	0.78 (0.16)
Koller, 2005 [24]	Women	1.01 (0.12)	0.99 (0.11)	1.29 (0.13)	1.27 (0.12)
Zhang, 2005 [38]	Women	0.66 (0.12)	0.65 (0.11)	0.80 (0.14)	0.79 (0.14)
van Meurs, 2006 [39]	Men	0.92 (0.12)	0.91 (0.12)	1.17 (0.20)	1.14 (0.19)
	Women	0.83 (0.14)	0.83 (0.14)	1.04 (0.19)	1.02 (0.19)
Ezura, 2007 [18]	Women	-	-	0.91 (0.22)	0.88 (0.19)
Saarinen, 2007 [40]	Men	1.18 (0.15)	1.09 (0.14)	1.23 (0.13)	1.18 (0.14)
Giroux, 2007 [20]	Women	0.88 (0.14)	0.87 (0.14)	1.12 (0.17)	1.10 (0.17)
Grundberg, 2007 [21]	Men	0.83 (0.13)	0.82 (0.13)	1.14 (0.20)	1.14 (0.20)
	Men	0.69 (0.11)	0.69 (0.12)	0.95 (0.17)	0.94 (0.18)
	Men	1.16 (0.15)	1.17 (0.17)	1.24 (0.15)	1.22 (0.14)
Brixen, 2007 [36]	Men	-	-	1.08 (0.12)	1.07 (0.17)

Values are mean (standard deviation).

Women of Caucasian background appeared to have significantly higher relative frequency of the AA genotype than their Asian counterparts (70% vs. 55%) [18,20,24,37-39]; however, the observation was not found in men (75% vs. 70%).

### Association between LRP5 genotypes and BMD

As genotype VV was low in most populations (i.e., approximately 3.4%), data from the VV and AV genotypes were combined into one group which was then compared to the AA genotype. This approach of combination was also utilized in most primary studies.

#### Pooled effect size

##### Classical meta-analysis

**Figure 2:**
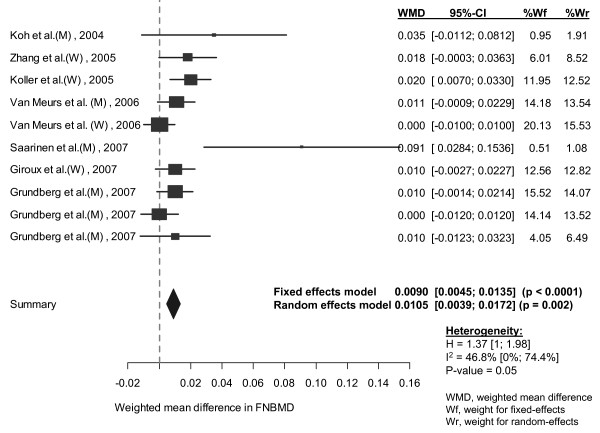
**Weighted mean difference and 95% confidence interval in femoral neck BMD between AA and AV/VV genotypes**. The size of plots was proportional to their sample size. Each study was shown difference of BMD in men (M) and women (W) using random effects model. The diamond showed the overall effect of the association. Reduced BMD was shown in group of AV/VV genotype compared to AA genotype when the diamond was set toward the right of the vertical line.

**Figure 3:**
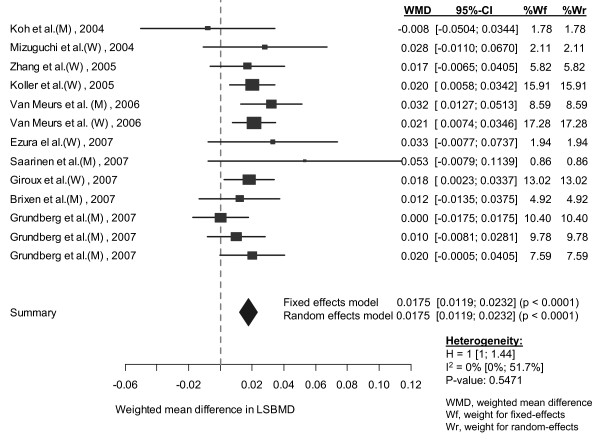
**Weighted mean difference and 95% confidence interval in lumbar spine BMD between AA and AV/VV genotypes**. Explanations were presented in figure 2.

In classical random-effects model, femoral neck BMD in individuals with genotype AA was significantly higher than in those with the AV and VV genotypes combined (WMD: 0.011, 95% CI: 0.004 to 0.017 g/cm^2^) (Table 3). Lumbar spine BMD in individuals homozygous for allele A was on average 0.018 g/cm^2 ^(95% CI: 0.012 to 0.023 g/cm^2^) higher than that in individuals with allele V (AV and VV genotypes combined) (Figure 2 and 3). For both sites, fixed-effects and random-effects analyses were almost identical.

**Table 3:** Sub-group analysis by sex and ethnicity (random-effects model)

Subgroup	Femoral neck BMD			Lumbar spine BMD		
	
	WMD (95% CI)	P value	*I*^2 ^(%)	WMD (95% CI)	P value	*I*^2 ^(%)
**Overall**	0.011 (0.004, 0.017)	0.002	46.8 (p = 0.05)	0.018 (0.012, 0.023)	<0.0001	0 (p = 0.55)
**Ethnicity**						
Asian	0.011 (-0.006, 0.028)	0.21	51.3 (p = 0.13)	0.014 (0.002, 0.027)	0.02	0 (p = 0.62)
Caucasian	0.011 (0.003, 0.019)	0.01	52.1 (p = 0.05)	0.018 (0.012, 0.025)	<0.0001	10.5 (p = 0.35)
**Gender**						
Men	0.011 (0.0004, 0.022)	0.04	50.6 (0.07)	0.014 (0.003, 0.025)	0.01	32.8 (p = 0.18)
Women	0.011 (0.001, 0.021)	0.03	55.8 (0.08)	0.020 (0.013, 0.028)	<0.0001	0 (p = 0.98)

WMD, weighted mean difference in BMD between AA and AV/VV genotypes

*I*^2^, inconsistent index

**Figure 4:**
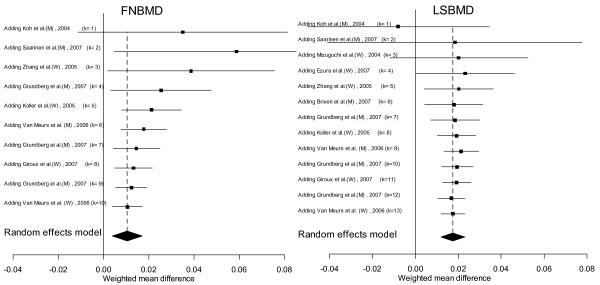
**Cumulative meta-analysis (by sample size) for femoral neck (left panel) and lumbar spine BMD (right panel)**. In each additional study, the mean difference (95% CI) of BMD difference between AA and AV/VV genotypes were computed accumulatively.

The cumulative meta-analysis showed that after a cumulative sample size of 15,285 individuals for femoral neck and 16,705 for lumbar spine, the association between A1330V variant and BMD became statistically apparent (Figure 4).

##### Bayesian meta-analysis

**Figure 5:**
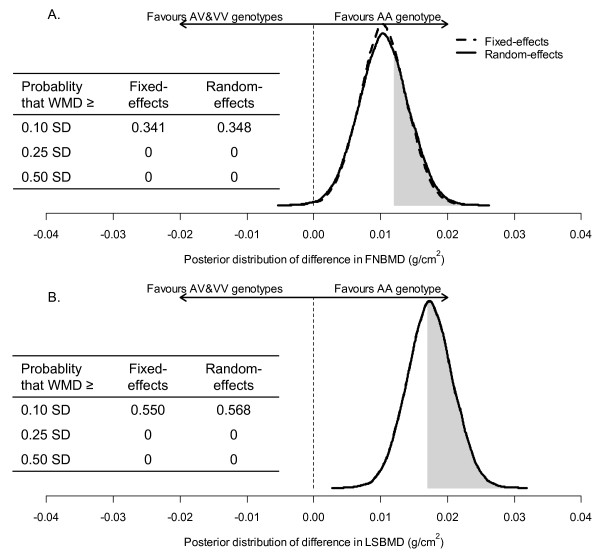
**Posterior distribution of weighted mean difference (WMD) in BMD at the femoral neck (A) and lumbar spine (B)**. For example, shaded areas in the figures represent for the probability that the effect size (AA vs. AV/VV) of >0.1 SD of FNBMD and LSBMD by using random-effects model, respectively. These areas accounted for ~34% of the whole area under the curve for femoral neck BMD and ~54% for lumbar spine BMD. In other words, the probability that the effect size (AA vs. AV/VV) of >0.1 SD was ~34% for FNBMD and ~54% for LSBMD. Results from fixed-effects and random-effects analysis were almost identical for LSBMD. FNBMD, femoral neck bone mineral density; LSBMD; lumbar spine bone mineral density; 1 SD was 0.12 g/cm^2 ^for BMD at the femoral neck and 0.17 g/cm^2 ^at the lumbar spine.

In Bayesian analysis, we analyzed two models separately: model I and model II. Model I included the data used for the "classical" analysis and in model II, we incorporated the data used for the "classical" analysis with recently published data [30]. The difference in results of two models was modest (data not shown); therefore, the results of model II were presented. Parameters obtained from Bayesian meta-analysis were used to estimate posterior distributions of mean difference in BMD between AA and AV/VV genotypes (Figure 5a and 5b). The area under the curve between any two points on the distribution is an estimate of the probability of effect size. For example, shaded areas in figures 5a and 5b represent for the probability that the effect size (AA vs. AV/VV) of >0.1 SD of FNBMD and LSBMD by using random-effects model, respectively (each SD was 0.12 g/cm^2^for FNBMD and 0.17 g/cm^2 ^for LSBMD). These areas accounted for ~34% of the whole area under the curve for femoral neck BMD and ~54% for lumbar spine BMD. In other words, the probability that the effect size (AA vs. AV/VV) of >0.1 SD was ~34% for FNBMD and ~54% for LSBMD. There was a 100% chance that the effect size was less than 0.25 SD. In other words, the probability for a possible difference in BMD between genotypes at both the femoral neck and lumbar spine was highly likely lower than 0.25 SD.

#### Subgroup analysis

In subgroup analyses, the effect of A1330V variant on BMD was found to be present in Asian populations at the lumbar spine (WMD between AA and AV/VV: 0.014; 95% CI: 0.002 to 0.027 g/cm^2^) and in Caucasian populations at either the lumbar spine (WMD: 0.018; 95% CI: 0.012 to 0.025 g/cm^2^) or at femoral neck (WMD: 0.011, 95% CI: 0.003 to 0.019 g/cm^2^) (Table 3). Analysis by sex revealed that the association between the A1330V variant and BMD was significant in both genders, with WMD for women being 0.02 (95% CI: 0.013 to 0.028 g/cm^2^) for lumbar spine and 0.011 (95% CI: 0.001 to 0.021 g/cm^2^) for femoral neck (Table 3). In men, WMD in BMD between AA and AV/VV were 0.014 (95% CI: 0.003 to 0.025 g/cm^2^) for lumbar spine and 0.011 (95% CI: 0.0004 to 0.022 g/cm^2^) for femoral neck (Table 3).

#### Assessment of heterogeneity and publication bias

There was no evidence of heterogeneity in lumbar spine BMD (I^2 ^= 0, p = 0.55). However, the effects of the A1330V variant on femoral neck BMD were significantly different among studies, with the coefficient of inconsistency being 46.8% (p = 0.05) (Table 3).

**Figure 6:**
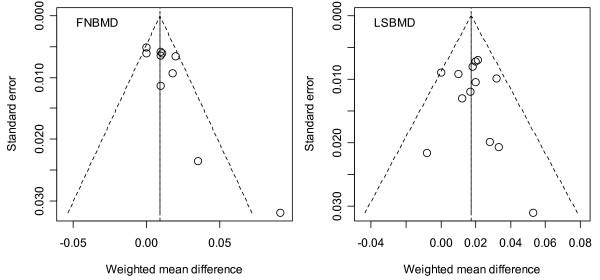
**Funnel plot of weighted mean difference for femoral neck BMD (p = 0.02) and for lumbar spine BMD (p = 0.65) versus inverse standard error Studies with higher effect size tended to have larger standard error.** (1 SD was estimated of 0.12 g/cm^2 ^and 0.15 g/cm^2 ^for femoral neck and lumbar spine, respectively).

In the funnel plot (Figure 6), there was symmetry in lumbar spine BMD (p = 0.65), suggesting no significant publication bias. However, the asymmetric feature in femoral neck BMD showed a trend of publication bias (p = 0.02). Nevertheless, when the analysis was limited to women only, there was no evidence of publication bias in either lumbar spine (p = 0.35) and femoral neck BMD (p = 0.17).

## Discussion

The discovery of linkage between the LRP5 gene and high bone mass was considered a genuine progress in the genetics of osteoporosis, a disorder that has been known to have a substantial genetic component. However, since linkage can only demonstrate a correlated transmission of alleles within pedigrees, the relative contribution of the LRP5 gene to BMD in population has to be assessed in association studies. Several association studies have tested the association between the LRP5 gene and BMD, but the results were inconsistent, with different polymorphisms being used and different study designs and sample sizes. In the present meta-analysis, by systematically combining all previous studies, there was a significant association between the A1330V polymorphism and lumbar spine BMD in both Asian and Caucasian populations. However, that variation at the A1330V polymorphism explained about 0.2 to 0.5% of between-subjects variation in BMD, which suggested that the effect of this LRP5 gene polymorphism on BMD was modest.

The present meta-analysis also suggests that the magnitude of effect of the A1330V on BMD was similar in both men and women, which was consistent with previous observations (Table 3) [17,39]. Similarly, the statistically significant association between A1330V and BMD at both sites was observed in Caucasian populations, but the association only found in Asian populations, which was likely due to the large sample size of the former populations.

It is interesting to observe that although the genotypic distribution was consistent with the Hardy-Weinberg's equilibrium law in most studies, the relative distribution of the A1330V genotypes varied remarkably between – as well as within-populations. For example, the AA genotype was 68% in Chinese women [38], 46% in Japanese women [18], or 73% of in Dutch women [39]. Among Caucasian populations, the AA genotype was detected in 56% in Dutch men [39] vs. 91% in Finnish men [40], and 76% in Danish men [36]. It is not clear why there was such a major difference in the genotypic distributions; however, population stratification and/or mixed ethnicities could be the underlying responsible factors. Gender and ethnicity may both interact to the influence of LRP5 polymorphism in association to BMD [41].

These analyses suggested that the clinical use of this gene variant was may be limited due to its modest effect size on BMD. The average difference in BMD between those homozygous for allele A and those with V allele was approximately 0.1 SD. Each SD lower BMD was associated with an approximately two-fold in fracture risk [1]. Therefore, it seems that the AA genotype within the A1330V polymorphism confers minimal protection against fracture via increased BMD. However, it is possible that the variant can have positive effect on fracture independent of BMD, and its use in conjunction with BMD and other clinical factors may identify a subset of high-risk individuals of fracture.

The magnitude of association between LRP5 polymorphism and BMD in this study was lower than that of between Collagen I alpha 1 gene (COLIA1) and BMD. In a previous meta-analysis, the difference between two homozygous genotypes of the COLIA1 gene was approximately 1 SD for femoral neck BMD (0.19 g/cm^2^, 95% CI 0.07 to 0.31), but not for lumbar spine BMD (0.09 g/cm^2^, 95% CI -0.03 to 0.21) [42]. On the other hand, a number of meta-analyses on the association between the vitamin D receptor gene (VDR) and BMD revealed an effect size of less than 0.1 SD [43,44].

A recent genome-wide association study between LRP5 polymorphisms and BMD or fracture in a large scale [45] found the A1330V polymorphism was associated with BMD at the lumbar spine and femoral neck, in which the risk allele V was associated with a 0.13 SD decrease in BMD, and accounted for 0.6% and 0.2% of the variation in lumbar spine and femoral neck BMD, respectively. Taken together, these results suggest that although there was a "true" association between the A1330V polymorphism and BMD, the effect is likely to be modest because the gene variant explained less than 1% of the variation in BMD.

The modest effect of the LRP5 gene on BMD is consistent with the view that the disorder is affected by several genes, each with small effect size [46]. Indeed, more than 50 genes have been proposed or identified to be associated with either BMD or fracture risk [47,48]; however, apart from the COLIA1, none of those genes have been conclusively demonstrated to have major effect on any osteoporosis-related phenotypes. The present meta-analysis suggested that the identification of specific genes that truly affect BMD can be a daunting task, because of a reliable result (i.e., low false positive rate), a typical association study requires at least 6600 individuals.

It is possible that the main effect of the LRP5 gene on BMD is modest, but it is also equally possible that the gene may confer a greater effect when it interacts with an environmental exposure or with other genes. However, these possibilities of gene-environment or gene-gene interactions have not been systematically explored in the field of osteoporosis. As a result, the present analysis can not address the issue of gene-environment or gene-gene interactions.

Although the present analysis supported an association between the LRP5 gene and BMD, the result must be considered in terms of a number of strengths and caveats. One strength of meta-analysis is that it increases the power for defining a fine association that is not usually possible in small individual studies. Nevertheless, given the weak association observed here, the possibility of false positive finding (or, for that matter, false negative finding) can not be ruled out, particularly in relation to the association found in men. Moreover, as with any meta-analysis, exclusion of pertinent unpublished studies is always a "threat" to the validity of the analysis.

The use of the Bayesian approach in this analysis deserves a mention. Tradition meta-analysis can estimate an effect size, but it is not possible to make a probabilistic statement about the effect size. In contrast, by treating the effect size as a random variable, Bayesian analysis can make inference on various probable effect sizes. Indeed, by combining a prior distribution with observed data from primary studies within the Bayesian theorem it is possible to make such an inference [47-49]. Thus, the Bayesian approach allows us to directly address the clinical question of "given the observed data, what is the probability of the 'true' difference in BMD between genotypes". This is different from the classical statistical approach in which the interpretation is entirely dependent on the p-value. The p-value is the probability that the test statistic is "significant" given that there is no difference in BMD between genotypes. This p-value based inference has been charged as counter-intuitive and even "false reasoning" [50]. In other words, classical statistical inference is concerned with the probabilistic behavior of a test statistic, whereas Bayesian inference is concerned with the research question. Moreover, In Bayesian analysis, one is not limited to consider the present data, but must take into account any data that have been previously been known. By using the Bayesian approach, we have shown that the difference in BMD between AA and AV/VV genotypes is less than 0.25 SD; there was virtually no chance that the effect size is greater than 0.25 SD.

In this analysis, there is no evidence of publication bias for the association between lumbar BMD and LRP5 gene. Although the best attempt has been made to search all relevant published studies, it is impossible to know how many "negative studies" are unpublished. Of the 14 eligible studies, it was only possible to retrieve analyzable data from 10 studies despite many efforts. The remaining 4 studies reported a non-significant association between the A1330V polymorphism and BMD [17,19,41,51]. Therefore, it could be argued that results of the present study may overestimate the true effect of LRP5 gene on BMD.

It should be noted that the present analysis was limited to a single SNP (rs3736228) within the LRP5 gene. Although this SNP has been used in most studies, other SNPs have also been shown to be associated with BMD. In recent years, the analysis of genetic association has shifted from the single SNP-based analysis to a more reductionistic approach such as SNP-based haplotypes. Some studies found no association between the LRP5 gene and BMD when analyzing each SNP as a separate variable, but were able to detect an association when the analysis was based on the use of SNP-based haplotypes [17,41]. The present meta-analysis did not consider the haplotype association; therefore, the estimate does not reflect any more complex association between the LRP5 gene and BMD. However, haplotype analyses reported in primary studies also showed that the magnitude of association between the gene and BMD is modest, with variation among haplotypes accounting for between 0.5 and 1.2% of the variation in BMD [22,52,53].

Thus, irrespective of the haplotype or single SNP-based analysis, results of this meta-analysis indicated that the A1330V variant within the LRP5 gene is modestly associated with bone mineral density, and that the modest effect size may limit its use in clinical setting.

## Abbreviations

All abbreviations are defined in the text.

## Competing interests

Dr John Eisman serves as a consultant and receives corporate appointment from Amgen, deCode, Eli Lilly and Company, GE-Lunar, Merck Sharp & Dohme Ltd., Novartis, Organon, Roche-GSK, sanofi-aventis and Servier. All other authors have neither financial nor non-financial competing interests that may be affected from the publication of the manuscript.

The first author is a recipient of the Harvey Carey Memorial Scholarship of the University of New South Wales for the postgraduate candidate. The second author is a recipient of a postdoctoral fellowship from the AMBeR (Australian Medical Bioinformatics Resource).

## Authors' contributions

BNHT and NDN obtained and analysed data. The manuscript was initially drafted by BNHT and revised by TVN and NDN. TVN, NDN and JAE were involved in the study design, data analysis, and in the conceptual discussion of the project. All authors contributed to the last version of the manuscript.

## Pre-publication history

The pre-publication history for this paper can be accessed here:



## References

[B1] Nguyen T, Sambrook P, Kelly P, Jones G, Lord S, Freund J, Eisman J (1993). Prediction of osteoporotic fractures by postural instability and bone density. BMJ.

[B2] Kanis JA, Gluer CC (2000). An update on the diagnosis and assessment of osteoporosis with densitometry. Committee of Scientific Advisors, International Osteoporosis Foundation. Osteoporos Int.

[B3] Marshall D, Johnell O, Wedel H (1996). Meta-analysis of how well measures of bone mineral density predict occurrence of osteoporotic fractures. BMJ.

[B4] Eddy DM, Johnston CC, Cummings SR, Dawson-Hughes B, Lindsay R, Melton LJI, Slemenda CW (1998). Osteoporosis: review of the evidence for prevention, diagnosis and treatment and cost-effectiveness analysis. Osteoporosis Int.

[B5] Browner WS, Seeley DG, Vogt TM, Cummings SR (1991). Non-trauma mortality in elderly women with low bone mineral density. Study of Osteoporotic Fractures Research Group. Lancet.

[B6] Kanis JA, Melton LJ, Christiansen C, Johnston CC, Khaltaev N (1994). The diagnosis of osteoporosis. J Bone Miner Res.

[B7] Nguyen TV, Livshits G, Center JR, Yakovenko K, Eisman JA (2003). Genetic determination of bone mineral density: evidence for a major gene. J Clin Endocrinol Metab.

[B8] Nguyen TV, Howard GM, Kelly PJ, Eisman JA (1998). Bone mass, lean mass, and fat mass: same genes or same environments?. Am J Epidemiol.

[B9] Nguyen TV, Eisman JA (2006). Pharmacogenomics of osteoporosis: opportunities and challenges. J Musculoskelet Neuronal Interact.

[B10] Gong Y, Vikkula M, Boon L, Liu J, Beighton P, Ramesar R, Peltonen L, Somer H, Hirose T, Dallapiccola B, De Paepe A, Swoboda W, Zabel B, Superti-Furga A, Steinmann B, Brunner HG, Jans A, Boles RG, Adkins W, Boogaard MJ van den, Olsen BR, Warman ML (1996). Osteoporosis-pseudoglioma syndrome, a disorder affecting skeletal strength and vision, is assigned to chromosome region 11q12-13. Am J Hum Genet.

[B11] Johnson ML, Gong G, Kimberling W, Recker SM, Kimmel DB, Recker RB (1997). Linkage of a gene causing high bone mass to human chromosome 11 (11q12-13). Am J Hum Genet.

[B12] Gong Y, Slee RB, Fukai N, Rawadi G, Roman-Roman S, Reginato AM, Wang H, Cundy T, Glorieux FH, Lev D, Zacharin M, Oexle K, Marcelino J, Suwairi W, Heeger S, Sabatakos G, Apte S, Adkins WN, Allgrove J, Arslan-Kirchner M, Batch JA, Beighton P, Black GC, Boles RG, Boon LM, Borrone C, Brunner HG, Carle GF, Dallapiccola B, De Paepe A, Floege B, Halfhide ML, Hall B, Hennekam RC, Hirose T, Jans A, Juppner H, Kim CA, Keppler-Noreuil K, Kohlschuetter A, LaCombe D, Lambert M, Lemyre E, Letteboer T, Peltonen L, Ramesar RS, Romanengo M, Somer H, Steichen-Gersdorf E, Steinmann B, Sullivan B, Superti-Furga A, Swoboda W, Boogaard MJ van den, Van Hul W, Vikkula M, Votruba M, Zabel B, Garcia T, Baron R, Olsen BR, Warman ML (2001). LDL receptor-related protein 5 (LRP5) affects bone accrual and eye development. Cell.

[B13] Little RD, Carulli JP, Del Mastro RG, Dupuis J, Osborne M, Folz C, Manning SP, Swain PM, Zhao SC, Eustace B, Lappe MM, Spitzer L, Zweier S, Braunschweiger K, Benchekroun Y, Hu X, Adair R, Chee L, FitzGerald MG, Tulig C, Caruso A, Tzellas N, Bawa A, Franklin B, McGuire S, Nogues X, Gong G, Allen KM, Anisowicz A, Morales AJ, Lomedico PT, Recker SM, Van Eerdewegh P, Recker RR, Johnson ML (2002). A mutation in the LDL receptor-related protein 5 gene results in the autosomal dominant high-bone-mass trait. Am J Hum Genet.

[B14] Boyden LM, Mao J, Belsky J, Mitzner L, Farhi A, Mitnick MA, Wu D, Insogna K, Lifton RP (2002). High bone density due to a mutation in LDL-receptor-related protein 5. N Engl J Med.

[B15] Van Wesenbeeck L, Cleiren E, Gram J, Beals RK, Benichou O, Scopelliti D, Key L, Renton T, Bartels C, Gong Y, Warman ML, De Vernejoul MC, Bollerslev J, Van Hul W (2003). Six novel missense mutations in the LDL receptor-related protein 5 (LRP5) gene in different conditions with an increased bone density. Am J Hum Genet.

[B16] Bollerslev J, Wilson SG, Dick IM, Islam FM, Ueland T, Palmer L, Devine A, Prince RL (2005). LRP5 gene polymorphisms predict bone mass and incident fractures in elderly Australian women. Bone.

[B17] Ferrari SL, Deutsch S, Choudhury U, Chevalley T, Bonjour JP, Dermitzakis ET, Rizzoli R, Antonarakis SE (2004). Polymorphisms in the low-density lipoprotein receptor-related protein 5 (LRP5) gene are associated with variation in vertebral bone mass, vertebral bone size, and stature in whites. Am J Hum Genet.

[B18] Ezura Y, Nakajima T, Urano T, Sudo Y, Kajita M, Yoshida H, Suzuki T, Hosoi T, Inoue S, Shiraki M, Emi M (2007). Association of a single-nucleotide variation (A1330V) in the low-density lipoprotein receptor-related protein 5 gene (LRP5) with bone mineral density in adult Japanese women. Bone.

[B19] Ferrari SL, Deutsch S, Baudoin C, Cohen-Solal M, Ostertag A, Antonarakis SE, Rizzoli R, de Vernejoul MC (2005). LRP5 gene polymorphisms and idiopathic osteoporosis in men. Bone.

[B20] Giroux S, Elfassihi L, Cardinal G, Laflamme N, Rousseau F (2007). LRP5 coding polymorphisms influence the variation of peak bone mass in a normal population of French-Canadian women. Bone.

[B21] Grundberg E, Lau EM, Lorentzson M, Karlsson M, Holmberg A, Groop L, Mellstrom D, Orwoll E, Mallmin H, Ohlsson C, Ljunggren O, Akesson K (2007). Large-scale association study between two coding LRP5 gene polymorphisms and bone phenotypes and fractures in men. Osteoporos Int.

[B22] Koay MA, Woon PY, Zhang Y, Miles LJ, Duncan EL, Ralston SH, Compston JE, Cooper C, Keen R, Langdahl BL, MacLelland A, O'Riordan J, Pols HA, Reid DM, Uitterlinden AG, Wass JA, Brown MA (2004). Influence of LRP5 polymorphisms on normal variation in BMD. J Bone Miner Res.

[B23] Koh JM, Jung MH, Hong JS, Park HJ, Chang JS, Shin HD, Kim SY, Kim GS (2004). Association between bone mineral density and LDL receptor-related protein 5 gene polymorphisms in young Korean men. J Korean Med Sci.

[B24] Koller DL, Ichikawa S, Johnson ML, Lai D, Xuei X, Edenberg HJ, Conneally PM, Hui SL, Johnston CC, Peacock M, Foroud T, Econs MJ (2005). Contribution of the LRP5 gene to normal variation in peak BMD in women. J Bone Miner Res.

[B25] DerSimonian R, Laird N (1986). Meta-analysis in clinical trials. Control Clin Trials.

[B26] Normal ST (1999). Meta-analysis: formulating, evaluating, combining, and reporting. Stat Med.

[B27] Spiegelhalter DJ, Abrams KR, Myles JP (2004). Bayesian Approaches to Clinical Trials and Health-Care Evaluation.

[B28] Sutton AJ, Abrams KR, Jones DR, Sheldon TA, Song F (2000). Methods for Meta-Analysis in Medical Research.

[B29] R Development Core Team (2008). R: A Language and Environment for Statistical Computing. 270 edn.

[B30] van Meurs JB, Trikalinos TA, Ralston SH, Balcells S, Brandi ML, Brixen K, Kiel DP, Langdahl BL, Lips P, Ljunggren O, Lorenc R, Obermayer-Pietsch B, Ohlsson C, Pettersson U, Reid DM, Rousseau F, Scollen S, Van Hul W, Agueda L, Akesson K, Benevolenskaya LI, Ferrari SL, Hallmans G, Hofman A, Husted LB, Kruk M, Kaptoge S, Karasik D, Karlsson MK, Lorentzon M, Masi L, McGuigan FE, Mellstrom D, Mosekilde L, Nogues X, Pols HA, Reeve J, Renner W, Rivadeneira F, van Schoor NM, Weber K, Ioannidis JP, Uitterlinden AG (2008). Large-scale analysis of association between LRP5 and LRP6 variants and osteoporosis. JAMA.

[B31] Spiegelhalter DJ, Thomas A, Best NJ, Lunn D (2003). WinBUGS User Manual Version 1.4. MRC Biostatistics Unit.

[B32] Cochran WG (1954). The combination of estimates from different experiments. Biometrics.

[B33] Higgins JP, Thompson SG (2002). Quantifying heterogeneity in a meta-analysis. Stat Med.

[B34] Macaskill P, Walter SD, Irwig L (2001). A comparison of methods to detect publication bias in meta-analysis. Stat Med.

[B35] Sterne JA, Gavaghan D, Egger M (2000). Publication and related bias in meta-analysis: power of statistical tests and prevalence in the literature. J Clin Epidemiol.

[B36] Brixen K, Beckers S, Peeters A, Piters E, Balemans W, Nielsen TL, Wraae K, Bathum L, Brasen C, Hagen C, Andersen M, Van Hul W, Abrahamsen B (2007). Polymorphisms in the low-density lipoprotein receptor-related protein 5 (LRP5) gene are associated with peak bone mass in non-sedentary men: results from the Odense androgen study. Calcif Tissue Int.

[B37] Mizuguchi T, Furuta I, Watanabe Y, Tsukamoto K, Tomita H, Tsujihata M, Ohta T, Kishino T, Matsumoto N, Minakami H, Niikawa N, Yoshiura K (2004). LRP5, low-density-lipoprotein-receptor-related protein 5, is a determinant for bone mineral density. J Hum Genet.

[B38] Zhang ZL, Qin YJ, He JW, Huang QR, Li M, Hu YQ, Liu YJ (2005). Association of polymorphisms in low-density lipoprotein receptor-related protein 5 gene with bone mineral density in postmenopausal Chinese women. Acta Pharmacol Sin.

[B39] van Meurs JB, Rivadeneira F, Jhamai M, Hugens W, Hofman A, van Leeuwen JP, Pols HA, Uitterlinden AG (2006). Common genetic variation of the low-density lipoprotein receptor-related protein 5 and 6 genes determines fracture risk in elderly white men. J Bone Miner Res.

[B40] Saarinen A, Valimaki VV, Valimaki MJ, Loyttyniemi E, Auro K, Uusen P, Kuris M, Lehesjoki AE, Makitie O (2007). The A1330V polymorphism of the low-density lipoprotein receptor-related protein 5 gene (LRP5) associates with low peak bone mass in young healthy men. Bone.

[B41] Xiong DH, Lei SF, Yang F, Wang L, Peng YM, Wang W, Recker RR, Deng HW (2007). Low-density lipoprotein receptor-related protein 5 (LRP5) gene polymorphisms are associated with bone mass in both Chinese and whites. J Bone Miner Res.

[B42] Mann V, Ralston SH (2003). Meta-analysis of COL1A1 Sp1 polymorphism in relation to bone mineral density and osteoporotic fracture. Bone.

[B43] Uitterlinden AG, Ralston SH, Brandi ML, Carey AH, Grinberg D, Langdahl BL, Lips P, Lorenc R, Obermayer-Pietsch B, Reeve J, Reid DM, Amedei A, Bassiti A, Bustamante M, Husted LB, Diez-Perez A, Dobnig H, Dunning AM, Enjuanes A, Fahrleitner-Pammer A, Fang Y, Karczmarewicz E, Kruk M, van Leeuwen JP, Mavilia C, van Meurs JB, Mangion J, McGuigan FE, Pols HA, Renner W, Rivadeneira F, van Schoor NM, Scollen S, Sherlock RE, Ioannidis JP (2006). The association between common vitamin D receptor gene variations and osteoporosis: a participant-level meta-analysis. Ann Intern Med.

[B44] Fang Y, Rivadeneira F, van Meurs JB, Pols HA, Ioannidis JP, Uitterlinden AG (2006). Vitamin D receptor gene BsmI and TaqI polymorphisms and fracture risk: a meta-analysis. Bone.

[B45] Richards JB, Rivadeneira F, Inouye M, Pastinen TM, Soranzo N, Wilson SG, Andrew T, Falchi M, Gwilliam R, Ahmadi KR, Valdes AM, Arp P, Whittaker P, Verlaan DJ, Jhamai M, Kumanduri V, Moorhouse M, van Meurs JB, Hofman A, Pols HAP, Hart D, Zhai G, Kato BS, Mullin BH, Zhang F, Deloukas P, Uitterlinden AG, D ST (2008). Bone mineral density, osteoporosis, and osteoporotic fractures: a genome-wide association study. The Lancet.

[B46] Nguyen TV, Blangero J, Eisman JA (2000). Genetic epidemiological approaches to the search for osteoporosis genes. J Bone Miner Res.

[B47] Xiong DH, Shen H, Zhao LJ, Xiao P, Yang TL, Guo Y, Wang W, Guo YF, Liu YJ, Recker RR, Deng HW (2006). Robust and comprehensive analysis of 20 osteoporosis candidate genes by very high-density single-nucleotide polymorphism screen among 405 white nuclear families identified significant association and gene-gene interaction. J Bone Miner Res.

[B48] Huang QY, Recker RR, Deng HW (2003). Searching for osteoporosis genes in the post-genome era: progress and challenges. Osteoporos Int.

[B49] Spiegelhalter DJ, Freedman LS, Parmar MKB (1994). Bayesian approaches to randomized trials. J R Stat Soc.

[B50] Diamond GA, Kaul S (2004). Prior convictions: Bayesian approaches to the analysis and interpretation of clinical megatrials. J Am Coll Cardiol.

[B51] Kiel DP, Ferrari SL, Cupples LA, Karasik D, Manen D, Imamovic A, Herbert AG, Dupuis J (2007). Genetic variation at the low-density lipoprotein receptor-related protein 5 (LRP5) locus modulates Wnt signaling and the relationship of physical activity with bone mineral density in men. Bone.

[B52] Albagha OM, Tasker PN, McGuigan FE, Reid DM, Ralston SH (2002). Linkage disequilibrium between polymorphisms in the human TNFRSF1B gene and their association with bone mass in perimenopausal women. Hum Mol Genet.

[B53] Rubin LA, Hawker GA, Peltekova VD, Fielding LJ, Ridout R, Cole DE (1999). Determinants of peak bone mass: clinical and genetic analyses in a young female Canadian cohort. J Bone Miner Res.

